# Relational dynamics associated with adolescent and young adult (13 to 23 years of age) partner violence: The role of inter-parental violence and child abuse

**DOI:** 10.1371/journal.pone.0283175

**Published:** 2023-12-28

**Authors:** Priya Maurya, T. Muhammad, Chanda Maurya

**Affiliations:** 1 Department of Population and Development, International Institute for Population Sciences, Mumbai, Maharashtra, India; 2 Department of Family and Generations, International Institute for Population Sciences, Mumbai, Maharashtra, India; 3 Department of Survey Research and Data Analytics, International Institute for Population Sciences, Mumbai, Maharashtra, India; University of Colombo Faculty of Medicine, SRI LANKA

## Abstract

**Purpose:**

The study aimed to examine the effect of witnessing inter-parental violence and experiencing childhood abuse on victimization of intimate partner violence (IPV) after marriage among adolescent and young girls.

**Method:**

Data were drawn from the second wave of the Understanding the lives of adolescents and young adults (UDAYA) survey (2018–2019). The sample size was 5480 married adolescent and young girls aged 13–23 years. The outcome variable of the study was the victimization of IPV. Descriptive statistics, bivariate analysis and structural equation modelling (SEM) were performed.

**Result:**

A total of 39% of married adolescent and young girls experienced physical violence, followed by sexual violence (35%) and emotional violence (28%) by their partner. Around 30% of respondents witnessed inter-parental violence, and 32% of the participants were beaten by their parents during childhood. Participants who had witnessed inter-parental violence were significantly correlated with experiencing childhood abuse, and this association was positively correlated with exposure to IPV in adolescence and young adulthood. Further, the parameter estimates of the indicators of IPV were highest for emotional violence (1.10) followed by physical violence (1.00) and sexual violence (0.62). Witnessing inter-parental violence significantly increases parents’ physical violence to adolescents and young adult girls (β = 0.49, P<0.001, CI: 0.47–0.51). No tie between witnessing inter-parental violence and childhood abuse mediates their effect on later victimization of IPV.

**Conclusion:**

The findings indicate that witnessing inter-parental violence is a strong risk factor for IPV victimization among adolescent and young adult girls. Our findings advocate prerequisite collaborative effort with multiple service providers for greater empowerment at national, state, community, and family levels to achieve SDG goals pertaining to eliminating violence against women.

## Introduction

Intimate partner violence (IPV) accounts for the largest proportion of violence against women, and an estimated 30% of women worldwide are likely to experience IPV in their lifetime [1]. Violence in the early aged (adolescence and young adulthood), affects women, with 24% of women in their adolescence and 26% of women in their young adulthood having already experienced violence at least once since the age of 15 years. Regional variations exist, with low-income countries reporting higher lifetime and, even more pronouncedly, higher past year experience of domestic violence compared with high-income countries [2]. More than 30% (CI: 22.6–39.5) of women reported IPV in south Asia [3], and the prevalence varied by countries [4]. In India, two out of five currently married women reported experiencing sexual, physical, or emotional violence from their intimate partners [5].

Importantly, existing evidence highlights the childhood and family experiences as victim-related psychological mechanisms that may explain the risk for IPV victimization. According to the systems theory [6], ‘one part of the family cannot be understood in isolation from the rest of the system, and what happens to one part of the system affects the entire family’ suggesting that families influence the behavior of its members. Inter-generational transmission of IPV is an important area of research, and several studies have explored the linkage among experiencing violence during childhood, parental violence and perpetration of IPV during adulthood [7–9]. Evidence of intergenerational transmission of IPV has also been supported by several cross-cultural studies [10–13], suggesting that past experiences of child abuse, current parental violence may predict the adolescents’ and young women’s experiences of IPV.

A growing body of literature suggests that IPV often affects children and adolescents in a household, either as indirect, when witnessing parental violence and direct as a victim who suffers from physical and/or sexual and/or psychological abuse [14–16]. A study among Brazilian adults found that regardless of being a victim of physical violence during childhood, witnessing parental violence was associated with being a victim of IPV in adult life, but not with becoming a perpetrator [17]. Individuals who experienced child abuse were more likely to perpetrate IPV and witnessing IPV between parents was associated with an increased frequency of IPV [15]. In multiple studies it is generally reported that both direct and indirect violent victimization may increase the risk of engaging in IPV during adulthood [12, 17–19].

**Fig 1:**
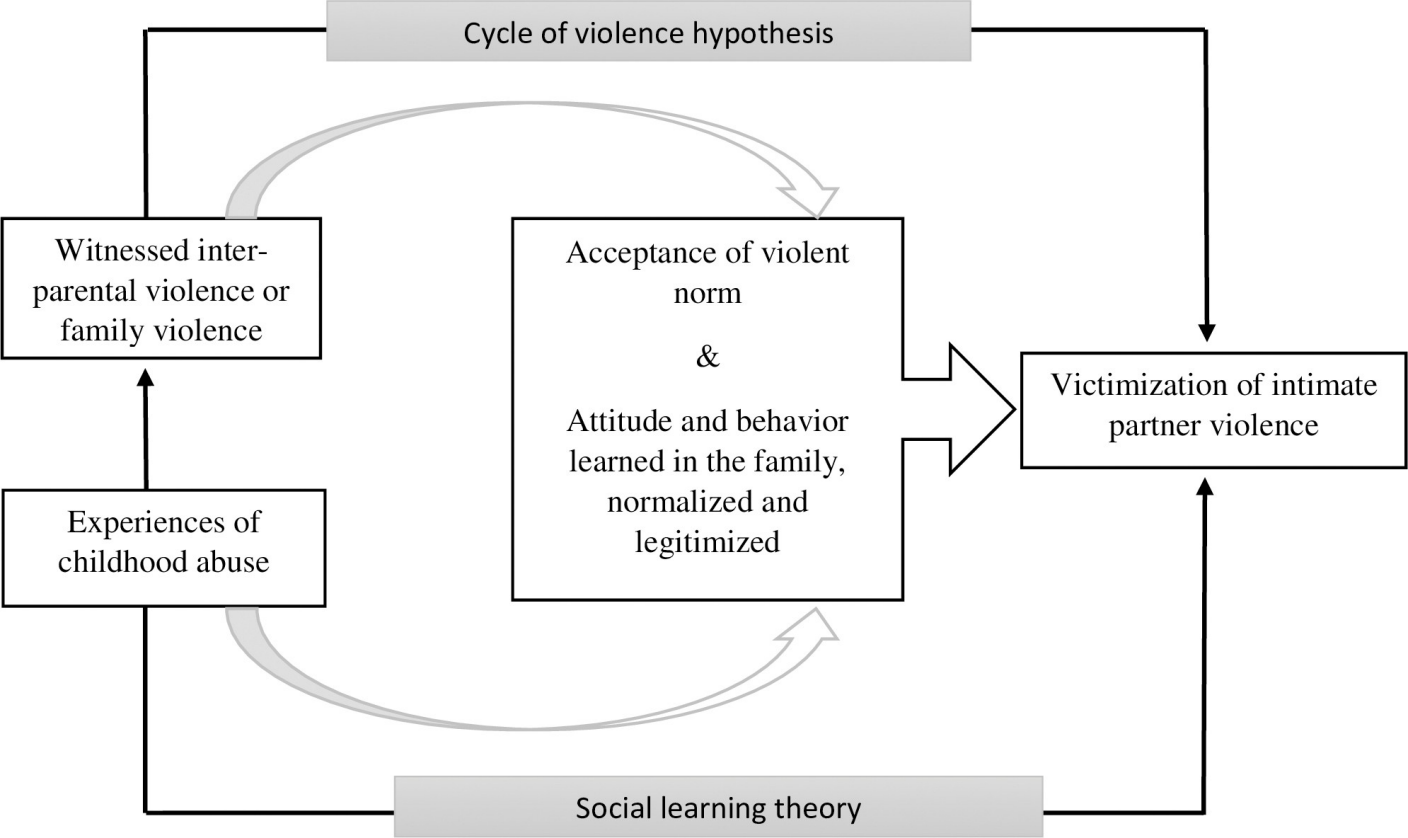
Conceptual framework for witnessing inter-parental violence, experiencing childhood abuse and exposure to IPV.

The cycle of violence hypothesis suggests that the experience of physical abuse raises individual’s risk for developing chronic aggressive behavior problems that leads to a cycle of violence [20, 21]. Similarly, Akers’ (1973) [22] social learning theory predicts that the prevalence of partner violence is greater among those who have witnessed others they admire using aggression [23]. Besides, experience of violence from other family members was positively associated several types of IPV in the past year [24]. Furthermore, factors such as early age at first marriage, ever having a terminated pregnancy, husband’s controlling behaviors, depressive symptoms and having an alcoholic husband were also found as risk factors for IPV among young women in earlier studies [13, 25, 26]. Poor socioeconomic status and acceptance of IPV in close family relationships also increased the odds of experiencing IPV among women [27–29]. Conceptual framework for witnessing inter-parental violence, experiencing childhood abuse and exposure to IPV are presented in **Fig 1**.

However, the general understanding on the linkage between witnessing inter-parental violence, experiencing childhood abuse and exposure to partner violence among young women remains limited in specific socio-cultural setting of India. One of the crucial aspects of gender equality and women/ girls’ empowerment as a major component of the Sustainable Development Goals (SDGs) is addressing partner violence, and India is falling behind to meet this goal. Thus, using a large representative sample of adolescent and young adult females from two major states of India, this study considers the question whether being exposed to child abuse or inter-parental violence increases the risk for victimization of violence after getting married among young women in India. The study hypothesis that there is significant effect of inter parental violence and child abuse on victimization of violence after marriage.

## Methods

### Data

Data were obtained from the ‘Understanding the lives of adolescents and young adults’ (UDAYA) project, survey conducted by the Population Council, New Delhi and funded by the Bill and Melinda Gates Foundation and the David and Lucile Packard Foundation. UDAYA is a longitudinal study conducted in the major states of Uttar Pradesh and Bihar in India following a cohort of adolescents aged 10–19 years [30].

For sampling at the base line (wave 1), the study used both cross-sectional and longitudinal designs and a multi-stage systematic sampling design was used. The UDAYA survey was designed to provide estimates at two time points, (i) at state level and (ii) at urban and rural areas level of the state. In the survey, 150 primary sampling units (PSUs), 75 for each residence separately, respondents were sampled in each state using Probability proportional to size (PPS) technique [30]. PSU list was stratified using four variables, (i) region (ii)village/ward size (iii) proportion of the population belonging to scheduled castes and scheduled tribes, (iv) female literacy. The household samples were selected on the basis of three stage sampling procedure in rural areas and four stage sampling procedure in urban areas [30].

Data collection for wave 1 was done during 2015–16 and after three years, wave 2 data were collected during 2018–19. In the current study, cross-sectional sample of only wave 2 is used, consisting information of married female adolescents aged 12–23 years. The total sample size for Uttar Pradesh and Bihar was 3600 and 2128 adolescents aged 12–23 years, respectively [30]. The sample size for this study was 5480 female adolescents who were married at the time of the survey.

### Measures

#### Outcome variable

Outcome variable of the study was exposure to IPV. Three indicators of IPV were included in the current study, namely physical violence, sexual violence and emotional violence. Physical violence was assessed by asking seven questions to the respondents, they were asked about physical hurt by husband, the questions included, (1) slap, (2) twist arm or pull hair, (3) push, shake or throw something, (4) punch, (5) kick, drag or beat, (6) try to choke or burn and (7) threaten or attack by knife, gun or other weapon in last 12 months. The responses to the above questions were “yes” and “no”. The scale of 0–14 point was then generated by additive method. The variable was then recoded into two categories; 0 “No (0–6)” and 1 “yes (7–14)” [31]. The categories were identified for analytical purpose. Sexual violence was assessed by asking a question i.e. husband ever forced you to have sex and categorised as “yes” and “no”. Emotional violence was assessed by asking a question i.e. husband ever do something to humiliate you or threatened you to hurt or harm someone close to you. The variable was categorised as “yes” and “no” [31].

#### Explanatory variables

Inter-parental violence was assessed using the question “Has your father ever beaten your mother?” It was coded as 1 “yes” if the respondent had an affirmative answer and otherwise 0 “no”. and physical violence by their parents was assessed using the direct question “Have you been physically hurt (for example, beaten) by your father or mother from the time you turned 10 years old?” the variable was recoded as 1 “yes” if the respondent physically hurt by their parent and otherwise 1 “no”. Individual factors: Age was coded as “13–17 years”, “18–20 years” and “21-23years”. Exposure to social media was coded as “no” and “yes”. Depressive symptoms were assessed by asking 9 questions from the respondents, the respondent was asked about the symptoms for past two weeks only. The questions included, (1) had trouble falling asleep or sleeping too much, (2) feeling tired or having little energy, (3) poor appetite or eating too much, (4) trouble concentrating on things, (5) had little interest or pleasure in doing things (6) feeling down, depressed or hopeless, (7) feeling bad about yourself, (8) been moving or speaking slowly, (9) had thoughts that respondent would be better off dead. All the above questions were asked on likert scale of four i.e., 0 “not at all”, 1 “less than once a week”, 2 “one week or more” and 3 “nearly every day”. The scale of 27 points was then generated using *egen* command in STATA 15 (Cronbach alpha: 0.86) [32, 33]. The variable was then recoded into three categories i.e., (i) Mild (0–9), (ii) Moderate (10–14) and (iii) Severe (15–27). Mild includes minimal and mild, moderate include moderate only and severe include moderately severe and severe. The categories were redefined for analytical purpose [30]. Substance use was categorized as “yes” if respondent use any tobacco product or alcohol and “no” otherwise. Adolescents co-resides with parents was coded as “both parents co-reside” and “anyone parent co-reside”. Educational level coded as “illiterate”, “primary and middle for up to 8 years of schooling” and “higher for 9 and more years of schooling” [34]. Paid work in past 12 month was coded as “no” and “yes”. Religion was coded as “Hindu” and “non-Hindu”. Caste was coded as 1“Scheduled Caste/Scheduled Tribe (SC/ST)”, 2“Other background class (OBC)” and 3“other”. Wealth index was coded as “Poor”, “Middle” and “Rich”. Place of residence was coded as “rural” and “urban” [35].

### Statistical analysis

Initially, descriptive statistics and bivariate analysis were performed to understand the characteristics of the study population. Further, SEM technique using the Maximum Likelihood estimation (MLE) procedure had applied to estimate the covariance matrix. For the specification of model, model-fit indices, statistical significance of the parameter estimated and also effect size and its direction used.

Model fit was examined using the guidelines [36] according to which good model fit is reached when chi-square value is low and non-significant; comparative fit index (CFI) values are 0.95 or more, and root mean square error of approximation (RMSEA) values are 0.05 or less (0.6–0.8 indicates a mediocre model fit). Chi-square difference testing and Akaike Information Criterion (AIC) was used to compare the models for the best fit whereby the lowest AIC indicated the best fitting model [37]. Statistically significant coefficients within the best fitting model were then examined for interpreting specific inter-spousal violence or IPV and children witnessing violence and child abuse.

## Results

**Fig 2:**
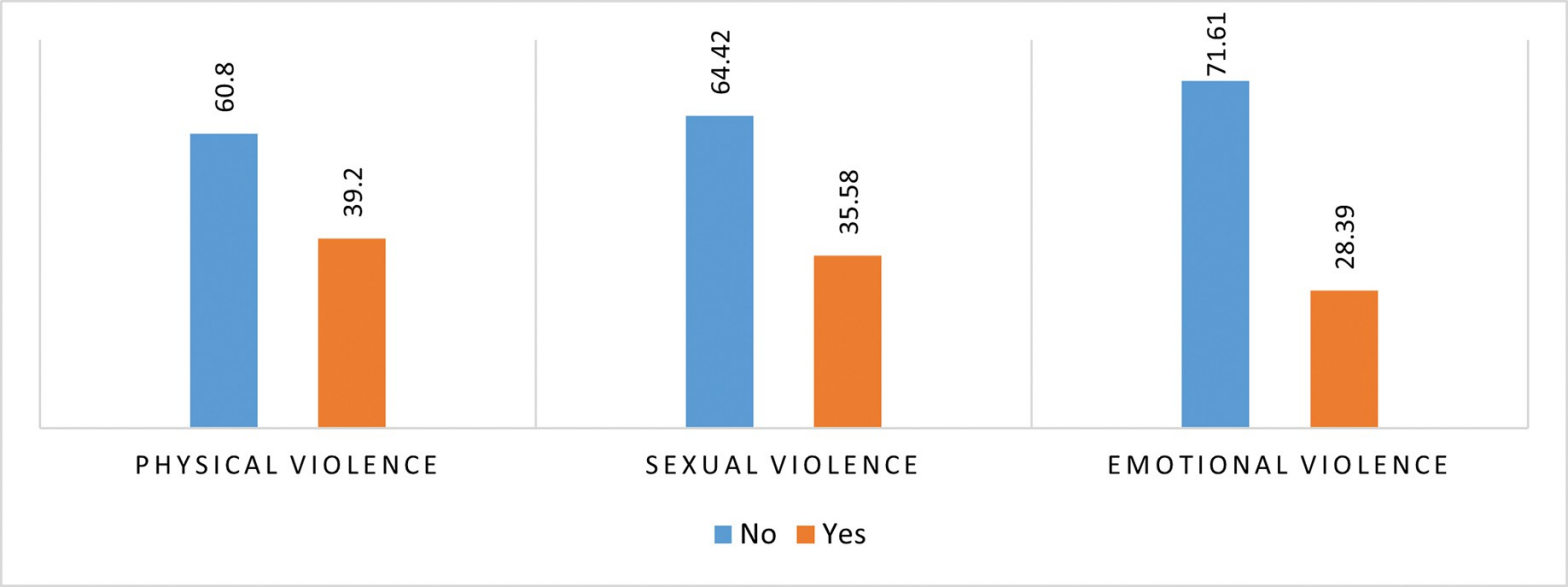
Percentage distribution of girls aged 13–23 years who experienced intimate partner violence.

**Fig 3:**
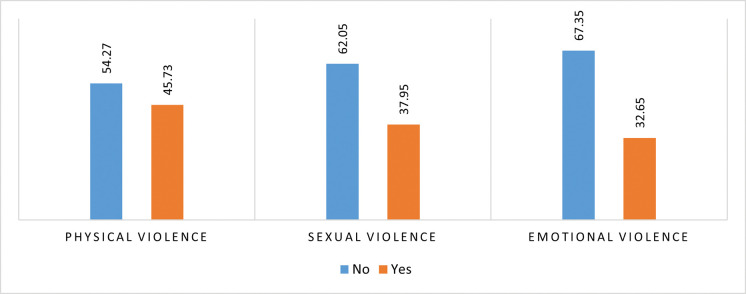
Percentage distribution of intimate partner violence among witnesses of inter-parental violence.

**Fig 4:**
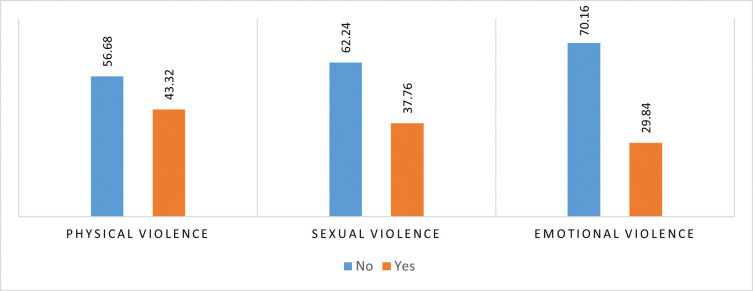
Percentage distribution of intimate partner violence among those experience physical violence by their parents.

**Fig 2** depicts the percentage distribution of married adolescent girls who had experienced different types of violence. About 39% of married adolescent girls experienced physical violence, followed by sexual violence (35%) and emotional violence (28%) by their partner. The percentage distribution of intimate partner violence among witnesses of inter-parental violence and also among those who experiences physical violence by their parents are presented in the **Figs 3** and **4** respectively. Nearly 46% of the married girls, who were witnesses of inter-parental violence and 43% of the married girls who were victims of parental violence, experienced physical violence. Nearly 2 out of five girls experience sexual violence by their partner, who were either witnesses of inter-parental violence or victim of violence by their parents in past. One third of the girls, witnesses of inter-parental violence, experiences emotional violence by their intimate partner.

The socioeconomic characteristics of the study population are presented in **Table 1**. Around 30% of respondents reported that they ever witnessed their father ever beating their mother. One-third of girls experienced physical violence from their parents during childhood. Nearly 36% of adolescents were in the age group of 18–20 years. The majority of the respondents were residing with their parents, mother and father. One-third of the respondents engaged in paid work. Only 17% of adolescents were exposed to social media, and nearly 5% of girls had severe depressive symptoms.

**Table 1:** Socio-demographic characteristics of adolescent girls and young women aged 13–23 years.

Background Characteristics	N	%
**Experienced inter-parental violence**		
No	3,993	69.71
Yes	1,735	30.29
**Experienced physical violence by parents**		
No	3,893	67.95
Yes	1,836	32.05
**Age**		
13–17	118	2.06
18–20	2076	36.24
21–23	3534	61.7
**Educational attainment**		
Never	1088	18.98
Up to secondary	2143	37.42
Higher	2497	43.6
**Parents co-residence**		
With single parent	247	4.3
With both parents	5482	95.7
**Paid work in last 12 month**		
No	3519	61.43
Yes	2209	38.57
**Social media exposure**		
No	4663	81.4
Yes	1066	18.6
**Depression**		
Mild/Minimal	4942	86.28
Moderate	512	8.94
Severe	274	4.78
**Substances use**		
No	5267	91.95
Yes	461	8.05
**Caste**		
SC/ST	1696	29.62
Non SC/ST	4032	70.38
**Religion**		
Hindu	4705	82.15
Muslim	1023	17.85
**Wealth index**		
Poor	2057	35.91
Middle	1304	22.76
Rich	2368	41.34
**Place of residence**		
Urban	675	11.78
Rural	5054	88.22
**State**		
Uttar Pradesh	3600	62.85
Bihar	2128	37.15
**Total**	**5480**	**100**

**Table 2** represents the correlation matrix, including different types of violence and key variables. Adolescent girls who had ever witnessed parental violence were significantly correlated with experiencing abuse against them by parents during childhood, and this association was positively correlated with the physical, sexual and emotional violence in adolescence.

**Table 2:** Bivariate correlations between outcome and key variables.

Correlation	Physical violence	Sexual violence	Emotional violence	Experienced inter-parental violence	Experienced physical violence by parents
**Physical violence**	1.00				
**Sexual violence**	0.288	1.000			
**Emotional violence**	0.544	0.330	1.000		
**Experienced inter-parental violence**	0.082	0.042	0.062	1.000	
**Experienced physical violence by parents**	0.055	0.030	0.019	0.482	1.000

### Model evaluation

**Fig 5:**
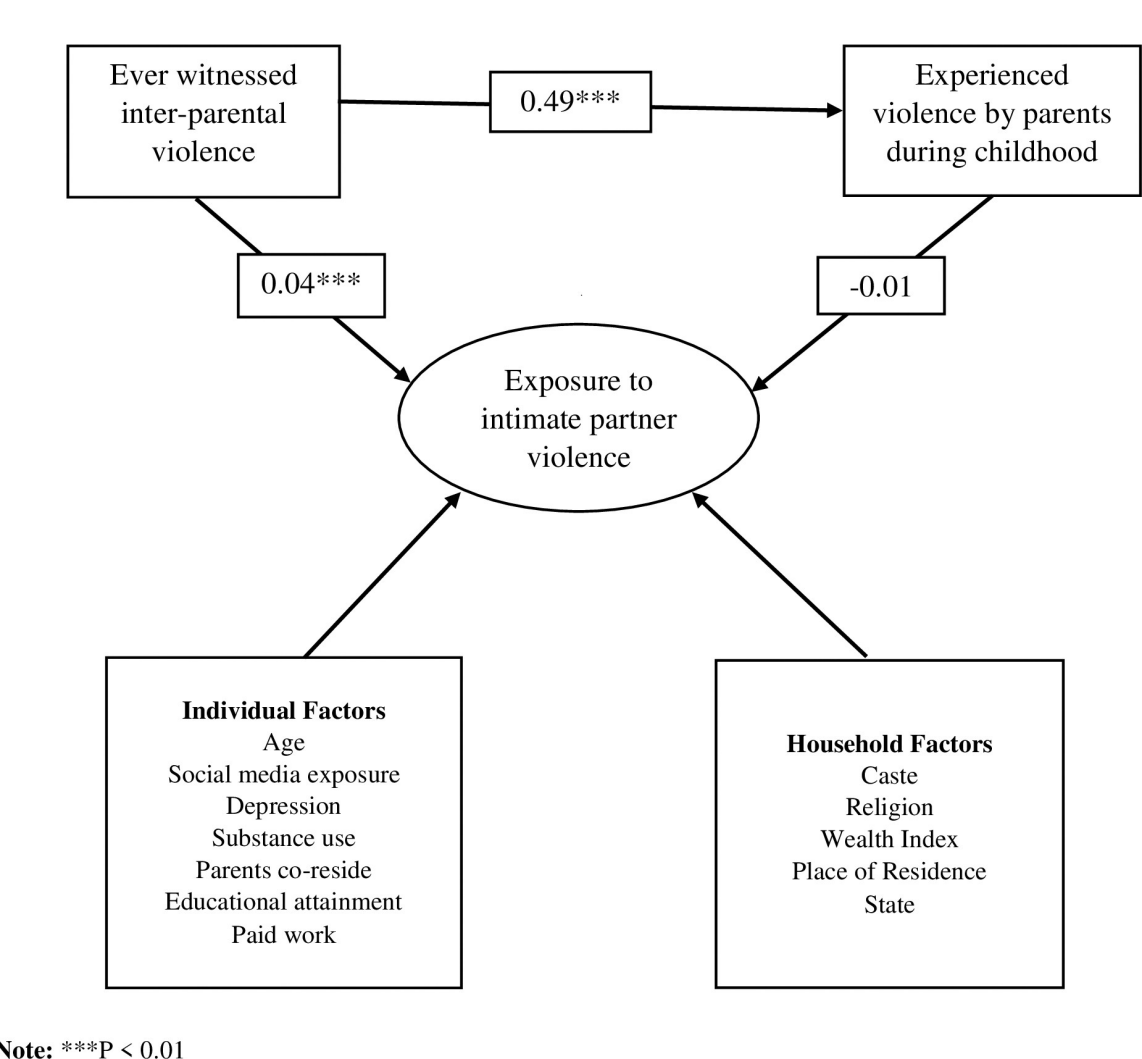
Structural equation modelling. Note: 1) Ovals show latent variables, 2) Boxes show observed variables, 3) Error terms are excluded for simplicity. **Note:** ***<P<0.01.

**Fig 5** and **Table 3** indicate the generated model with the standardized parameter estimates. The conditions of the goodness of fit of a model differ among authors, and the traditional method for absolute fit indices is chi-square. However, the present study had a large sample size; therefore, the traditional measure of RMSEA below 0.05 was used to indicate a good fit. Other fit indices, such as model parsimony and incremental fit indices, are sometimes considered when performing an EFA, which requires model modification. The global goodness-of-fit statistics showed a chi-square test statistic of 302.77, d.f. = 52, P < 0.001. The large absolute fit index (chi-square) was due to the large sample size in the research. Though the RMSEA, which is not sensitive to the sample size, indicating a good fit (RMSEA = 0.030), it was lower than the acceptable traditional level of 0.05.

**Table 3:** Multivariate regression coefficients (β), standard error (SE), and 95% confidence interval (CI) of the estimated structural equation model.

Variables	β(SE)	95%CI
**Experienced physical violence by parents**		
Experienced inter-parental violence	0.49(0.01)***	(0.47–0.51)
**Intimate partner violence**		
Experienced physical violence by parents	-0.01(0.01)	(-0.03–0.02)
Experienced inter-parental violence	0.04(0.01)***	(0.01–0.06)
Age: 18–20 years	0.05(0.03)	(-0.02–0.11)
Age: 21–23 years	0.07(0.03)**	(0–0.13)
Educational attainment: Up to secondary	-0.04(0.01)***	(-0.07 - -0.02)
Educational attainment: Higher	-0.13(0.01)***	(-0.16 - -0.1)
Parents co-residence: Residing with both parents	0.11(0.02)***	(0.06–0.16)
Paid work in last 12 month	0.04(0.01)***	(0.02–0.06)
Social Media exposure	-0.05(0.01)***	(-0.07 - -0.02)
Depression: Moderate	0.14(0.02)***	(0.11–0.18)
Depression: Severe	0.22(0.02)***	(0.17–0.27)
Substance use	0.06(0.02)***	(0.02–0.1)
Caste: Non ST/SC	-0.04(0.01)***	(-0.07 - -0.02)
Religion: Non Hindu	-0.02(0.01)	(-0.05–0.01)
Wealth quintile: Poor	0.06(0.01)***	(0.03–0.08)
Wealth quintile: Middle	0.02(0.01)	(-0.01–0.05)
Place of residence: Urban	-0.01(0.01)	(-0.04–0.01)
State: Bihar	0.16(0.01)***	(0.13–0.18)

**Note: ®:** Reference category; **SC/ST:** Schedule Tribe/ Schedule Caste; Significance level: **P<0.05, ***P < 0.01

### Measurement variables for intimate partner violence

All the measurement variables in the endogenous variables of IPV contributed considerably to the model and were statistically significant at P < 0.001. The parameter estimates of the indicators of the ‘IPV’ latent were the highest for emotional violence (1.1), followed by physical violence (1.0) and sexual violence (0.62) in **Table 4**.

**Table 4:** Multivariate parameter estimates (β), standard error (SE) and 95% confidence interval (CI) of the measurement variables in the structural equation model.

Codes	Indicators	Β(SE)	95%CI
**Violence**			
Physical violence	Physical violence	1.00	
Sexual violence	Sexual violence	0.62(0.02)***	(0.57–0.67)
Emotional violence	Emotional violence	1.09(0.04)***	(1.02–1.16)
Variance (e. Experienced physical violence by parents)		0.17(0)	(0.16–0.17)
Variance (e. Physical violence)		0.13(0)	(0.12–0.14)
Variance (e. Sexual violence)		0.2(0)	(0.19–0.21)
variance(e. Emotional violence)		0.09(0)	(0.08–0.1)
Variance (e. Intimate partner violence)		0.1(0)	(0.09–0.11)
**Model fit statistics**			
Chi-Square	0.00		
**RMSEA**	0.03		
CFI	0.95		
TLI	0.93		
SRMR	0.02		
CD	0.35		

***P < 0.001 and CD, coefficient of determination; CFI, Comparative Fit Index; RMSEA, Root Mean Square Error of Approximation; SRMR, Standardized Root Mean Square Residual; TLI, Tucker–Lewis index.

### Multivariate structural model analysis

In the observed pathway, there were significant linkages between witnessing inter-parental violence, experience of physical violence during childhood, and IPV. Inter-parental violence witnessing ever significantly increased parents’ physical violence during childhood among adolescent and young girls [β = 0.49, P<0.001], but witnessing parental physical violence did not quite relate to IPV. No tie between witnessing inter-parental violence and childhood abuse mediates their effect on later IPV. There was a direct and significant relationship between witnessing inter-parental violence at a younger age and IPV later [β = 0.04, P<0.001]. Adolescents in the older ages (21–23 years) were positively associated with IPV. Those adolescent girls who used substance [β = 0.06, P<0.001], resided with both parents [β = 0.11, P<0.001], engaged in paid work in last 12 months [β = 0.04, P<0.001], suffered moderate or severe depressive symptoms as well as belonged to poor wealth quintile [β = 0.06, P<0.001] were positively associated with victimization of IPV. However, adolescents whose educational level was up to secondary class or more, exposed to social media, [β = -0.05, P<0.001], belonged to non-SC/ST category [β = -0.04, P<0.001] and urban residence [β = -0.01, P<0.001] were negatively associated with victimization of IPV.

## Discussion

Existing research on IPV highlights that family violence leads to greater risk for adulthood family violence. The theory of cycle of violence also hypothesized that exposure to inter-parental violence or child abuse escalates to partner abuse victimization in adulthood [38]. While with India’s commitment to achieving SDGs with a focus on Goal 5.2 pertaining to eradication of all forms of violence against women and girls, it is important to understand the etiology of domestic abuse while having exposure to inter-parental violence or child abuse. In doing so, we attempted to address research gap related to whether being exposed to child abuse or inter-parental violence increases the risk for victimization of violence after getting married among adolescent girls in India.

The IPV is often convoluted in a nexus of family, social and cultural affairs and it has numerous implications on women, their families, and societies. With the patriarchal ideology in India, women are portrait as subordinate to men that may increase a women’s vulnerability for violence victimization [39, 40]. Taking this into account, the present study analysed married women only and found a higher rate of violence experienced by adolescent girls than the nationally representative estimates [41]. In accordance with previous studies [38, 42, 43], our study revealed that child beating is more customary in such households where women are abused. Childhood experience of inter-parental violence increases a person’s vulnerability to experience physical violence by parents. This may be suggested that children victimize abuse through the way of punishing or controlling the inter-parental violence. Also, frequency and severity of domestic violence concerning women is positively associated with mother’s and father’s physical aggression towards children in the household [44]. Previous studies also documented experience of childhood family abuse as a risk factor for intimate partner violence victimization [38, 45]. A systematic review on consequences of maltreatment of children’s lives reported that children who had been witnessed domestic violence in their childhood and had been victim of child abuse, were three times more likely to report IPV victimization in later life [43]. However, in present study there are negative association was observed between parents beaten respondents to victimization of violence in later life. This suggests that child abuse do not necessarily lead one’s risk for IPV.

Findings from the study further confirmed that adolescent girls who had documented history of inter parental violence were at higher risk of victim of IPV after getting married. This finding has been supported through a few studies where evidence indicated that witnessing parental violence in early age is linked with negative outcomes such as female victimization of intimate partner violence and male perpetration of violence in their adulthood [46, 47]. The study tracked to the possibility of intergenerational transmission of violence that supported when children witness family origin violence, they may justify or grow with the perception that these strategies are appropriate for solving the problem between couples. They may also consider behaving in similar way for solving their own problem and normalised the family violence [48]. The social learning theory also is in same line that children often idealized the communication style and behaviours of their surrounding peoples [40, 44]. Furthermore, a recent evidence from India indicate that 41.5% of woman’s believed that a man can abuse his wife under certain circumstances such as disobedience and neglect of household chores [40].

Contrary to the available literature [45, 46] that showed the occurrence of IPV decreases with age, this association has not found in the present study. One of the possible explanation for this lack of significant association between age and IPV victimization could be smaller number of cases in the sample in particular group. Our results pointed out that education has a protective effect on IPV risk. The literature identified education as a proxy of socioeconomic status [49], and economically weaker section reported higher prevalence of IPV [50, 51]. Women have greater personal skillset, knowledge and empowerment with advancement in education that could be the reason for lowering the reduction in IPV [40, 50].

Adolescent girls who are engaged paid work is positively associated with victimization of IPV. The possible reason could be that adolescent girls who are in paid work may belong to the lowest level of the economic strata and socially disadvantageous groups. Evidence suggested that lower socioeconomic status with poverty make them more vulnerable especially towards IPV [46]. Additionally, it is also possible that adolescent girls from higher household reports socially desirable response to questions related to IPV due to social stigma and cultural secrecy. A closer look at association between social media and victimization of IPV shows that exposure to social media decreases the magnitude of victimization of violence among adolescent girls. In recent years, the mass media strategies to address the issue of IPV have been increasing and important contributor for making progress in attitude towards IPV [52]. Such type of mechanism provides strength to women to curb violence against them. Depressive symptom is a crucial variable in the context of the present study. Unlike findings from the previous studies [51, 53], our study indicates that adolescent girls facing depression are negatively associated with victimization of IPV. This may suggest that poor mental health do not necessarily lead to an increased risk of experiencing IPV among adolescent girls or the result could be due to the limited number of cases in the total sample. Also, consistent with earlier evidence, the study noticed that substance users have a positive association with the victimization of IPV.

## Limitation

This present study has several drawbacks. First, there are high chances of under reporting of questions pertaining to violence due to social stigma associated with it. Again, the assessment of sexual and emotional violence using single-item questions are limiting and may result in reporting biases in this study. Second, this study included father beaten mother and children beaten by their parents which include only physical violence whereas emotional abuse in childhood is also potential risk factor for victimization of IPV. Third, the present study is based on quantitative data only not qualitative. Third, qualitative data may provide in depth details regarding familial cycle of violence from parents to children and specific issue related to intimate partner violence. Fourth, this study presents a snapshot of inter-parental violence and child abuse with victimization of IPV in later life only for one point of data collected.

## Conclusion

In summary, our findings are an initial step in uncovering some mediating factors linking witnessing inter-parental violence and experiencing violence during childhood by parents and victimization of IPV among adolescent and young adult girls. The findings indicate that witnessing inter-parental violence is a strong risk factor for IPV victimization among adolescent and young adult girls which is aligned with intergenerational transmission of violence theory. However, the experience of violence during childhood from parents is not associated with IPV victimization in this study, indicating that childhood abuse could be only one aspect of victimization of IPV. Our findings advocate prerequisite collaborative effort with multiple service providers for greater empowerment at national, state, community, and family levels to achieve SDG goals pertaining to eliminating violence against women. Interventions and policies related to IPV should not be concentrated on women empowerment only but also work with men and societies to connote gender norms, values, and equality. Additionally, education campaigns and behavioral programmes should be nourished from younger age for both girls and boys to transpose the social attitude towards IPV.

## References

[1] World Health Organization (WHO). *Intimate Partner Violence*. 2013. Epub ahead of print 2013. doi: 10.1016/B978-0-08-097086-8.35026–7

[2] Sardinha L, Catalán HEN. Attitudes towards domestic violence in 49 low-and middle-income countries: A gendered analysis of prevalence and country-level correlates. *PloS One* 2018; 13: e0206101. doi: 10.1371/journal.pone.0206101 30379891 PMC6209205

[3] Wang Y, Fu Y, Ghazi P, et al. Prevalence of intimate partner violence against infertile women in low-income and middle-income countries: a systematic review and meta-analysis. *Lancet Glob Health* 2022; 10: e820–e830. doi: 10.1016/S2214-109X(22)00098-5 35561719 PMC9115867

[4] Fulu E, Jewkes R, Roselli T, et al. Prevalence of and factors associated with male perpetration of intimate partner violence: findings from the UN Multi-country Cross-sectional Study on Men and Violence in Asia and the Pacific. *Lancet Glob Health* 2013; 1: e187–e207. doi: 10.1016/S2214-109X(13)70074-3 25104345

[5] Yoshikawa K, Agrawal NR, Poudel KC, et al. A lifetime experience of violence and adverse reproductive outcomes: Findings from population surveys in India. *Biosci Trends* 2012; 6: 115–121. doi: 10.5582/bst.2012.v6.3.115 22890159

[6] Whitchurch GG, Constantine LL. Systems Theory. In: Sourcebook of Family Theories and Methods. *A Contextual Approach*. 1993, pp. 167–170.

[7] Watt ME, Scrandis DA. Traumatic Childhood Exposures in the Lives of Male Perpetrators of Female Intimate Partner Violence. *J Interpers Violence* 2013; 28: 2813–2830. doi: 10.1177/0886260513488694 23708778

[8] Smith CA, Ireland TO, Park A, et al. Intergenerational continuities and discontinuities in intimate partner violence: A two-generational prospective study. *J Interpers Violence* 2011; 26: 3720–3752. doi: 10.1177/0886260511403751 21810795

[9] Askeland IR, Evang A, Heir T. Association of violence against partner and former victim experiences: A sample of clients voluntarily attending therapy. *J Interpers Violence* 2011; 26: 1095–1110. doi: 10.1177/0886260510368152 20587478

[10] Fehringer JA, Hindin MJ. Like Parent, Like Child: Intergenerational Transmission of Partner Violence in Cebu, the Philippines. *J Adolesc Health* 2009; 44: 363–371. doi: 10.1016/j.jadohealth.2008.08.012 19306795 PMC4181364

[11] Jin X, Eagle M, Yoshioka M. Early exposure to violence in the family of origin and positive attitudes towards marital violence: Chinese immigrant male batterers vs. controls. *J Fam Violence* 2007; 22: 211–222.

[12] Kerley KR, Xu X, Sirisunyaluck B, et al. Exposure to family violence in childhood and intimate partner perpetration or victimization in adulthood: Exploring intergenerational transmission in Urban Thailand. *J Fam Violence* 2010; 25: 337–347.

[13] Koenig MA, Stephenson R, Ahmed S, et al. Individual and Contextual Determinants of Domestic Violence in North India. 96. Epub ahead of print 2006. doi: 10.2105/AJPH.2004.050872 16317213 PMC1470450

[14] Warner TD, Swisher RR. The effect of direct and indirect exposure to violence on youth survival expectations. *J Adolesc Health* 2014; 55: 817–822. doi: 10.1016/j.jadohealth.2014.06.019 25204591

[15] Song A, Wenzel SL, Kim JY, et al. Experience of Domestic Violence During Childhood, Intimate Partner Violence, and the Deterrent Effect of Awareness of Legal Consequences. *J Interpers Violence* 2017; 32: 357–372. doi: 10.1177/0886260515586359 25976313

[16] Cohodes E, Hagan M, Narayan A, et al. Matched trauma: The role of parents’ and children’s shared history of childhood domestic violence exposure in parents’ report of children’s trauma-related symptomatology. *J Trauma Dissociation* 2016; 17: 81–96. doi: 10.1080/15299732.2015.1058878 26158778

[17] Madruga CS, Viana MC, Abdalla RR, et al. Pathways from witnessing parental violence during childhood to involvement in intimate partner violence in adult life: The roles of depression and substance use. *Drug Alcohol Rev* 2017; 36: 107–114. doi: 10.1111/dar.12514 28134495

[18] Hamby S, Finkelhor D, Turner H, et al. Children’s exposure to intimate partner violence and other family violence. *Juv Justice Bull* 2011; 1–12.

[19] Menard S, Weiss AJ, Franzese RJ, et al. Types of adolescent exposure to violence as predictors of adult intimate partner violence. *Child Abuse Negl* 2014; 38: 627–639. doi: 10.1016/j.chiabu.2014.02.001 24594015

[20] Widom CS. The cycle of violence. *JD Finkelman Child Abuse Ultidisciplinary Surv Short- Long-Term Eff* 1995; 154–159.

[21] Dodge KA, Bates JE, Pettit GS. Mechanisms in the cycle of violence. *Science* 1990; 250: 1678–1683. doi: 10.1126/science.2270481 2270481

[22] Akers RL. Deviant behavior: A social learning approach.

[23] Sellers CS, Cochran JK, Branch KA. Social Learning Theory and Partner Violence: A Research Note. *Deviant Behav* 2005; 26: 379–395.

[24] Ler P, Sivakami M, Monárrez-Espino J. Prevalence and Factors Associated With Intimate Partner Violence Among Young Women Aged 15 to 24 Years in India: A Social-Ecological Approach. *J Interpers Violence* 2020; 35: 4083–4116. doi: 10.1177/0886260517710484 29294780

[25] Capaldi DM, Knoble NB, Shortt JW, et al. A Systematic Review of Risk Factors for Intimate Partner Violence. *Partn Abuse* 2012; 3: 231–280. doi: 10.1891/1946-6560.3.2.231 22754606 PMC3384540

[26] Hong Le MT, Tran TD, Nguyen HT, et al. Early Marriage and Intimate Partner Violence Among Adolescents and Young Adults in Viet Nam. *J Interpers Violence* 2014; 29: 889–910. doi: 10.1177/0886260513505710 24366961

[27] Gracia E, Rodriguez CM, Martín-Fernández M, et al. Acceptability of Family Violence: Underlying Ties Between Intimate Partner Violence and Child Abuse. *J Interpers Violence* 2020; 35: 3217–3236. doi: 10.1177/0886260517707310 29294751

[28] Ackerson LK, Subramanian S V. State gender inequailtiy, socioeconomic status and intimate partner violence (IPV) in India: A multilevel analysis. *Aust J Soc Issues* 2008; 43: 81–102.

[29] Kiss L, Schraiber LB, Heise L, et al. Gender-based violence and socioeconomic inequalities: Does living in more deprived neighbourhoods increase women’s risk of intimate partner violence? *Soc Sci Med* 2012; 74: 1172–1179. doi: 10.1016/j.socscimed.2011.11.033 22361088

[30] Santhya KG, Acharya R, Pandey N, et al. *Understanding the lives of adolescents and young adults (UDAYA) in Bihar, India*. 2017.

[31] Srivastava S, Kumar P, Govindu M, et al. Dowry demand and other risk factors for intimate partner violence against adolescent girls: A cross-sectional study in India. 10.1371/journal.pone.0312341 PMC11501023 39446894

[32] Maurya C, Muhammad T, Maurya P, et al. The association of smartphone screen time with sleep problems among adolescents and young adults: cross-sectional findings from India. *BMC Public Health* 2022; 22: 1–11. 36064373 10.1186/s12889-022-14076-x PMC9444278

[33] Maurya P, Meher T, Muhammad T. Relationship between depressive symptoms and self-reported menstrual irregularities during adolescence: evidence from UDAYA, 2016. *BMC Public Health* 2022; 22: 1–9. 35422014 10.1186/s12889-022-13196-8 PMC9011997

[34] Maurya C, Muhammad T, Dhillon P, et al. The effects of cyberbullying victimization on depression and suicidal ideation among adolescents and young adults: a three year cohort study from India. *BMC Psychiatry* 2022; 22: 1–14. 36085004 10.1186/s12888-022-04238-x PMC9461154

[35] Muhammad T, Srivastava S, Kumar P, et al. What predicts the early sexual debut among unmarried adolescents (10–19 years)? Evidence from UDAYA survey, 2015–16. *PLoS ONE* 2021; 16: 1–15. doi: 10.1371/journal.pone.0252940 34111205 PMC8192016

[36] Kline RB. *Principles and practices of structural equation modelling 4th edition*. 2016.

[37] Kuha J. AIC and BIC: Comparisons of assumptions and performance. *Sociol Methods Res* 2004; 33: 188–229.

[38] Heyman RE, Slep AMS. Do Child Abuse and Interparental Violence Lead to Adulthood Family Violence? *J Marriage Fam* 2002; 64: 864–870.

[39] Batra R, Reio TG. Gender Inequality Issues in India. *Adv Dev Hum Resour* 2016; 18: 88–101.

[40] Murugan V, Khaoo Y-M, Termos M. Intimate Partner Violence Against Women in India: Is Empowerment a Protective Factor? *Glob Soc Welf* 2020; 8: 199–211.

[41] ICF & IIPS. *International Institute for Population Sciences*, National family health survey-4, 2015–2016. 2017.

[42] Guedes A, Mikton C. Examining the Intersections between Child Maltreatment and Intimate Partner Violence. *West J Emerg Med* 2013; 14: 377–379. doi: 10.5811/westjem.2013.2.16249 23997846 PMC3756703

[43] Fry D, Mccoy A, Swales D. The Consequences of Maltreatment on Children ‘ s Lives: A Systematic Review of Data From the East Asia and Pacific Region The Consequences of Maltreatment on Children ‘ s Lives: A Systematic Review of Data From the East Asia and Pacific Region. *TRAUMA VIOLENCE ABUSE* 2012; 13: 209–233. 22899705 10.1177/1524838012455873

[44] Temple JR, Shorey RC, Tortolero SR, et al. Importance of Gender and Attitudes about Violence in the Relationship between Exposure to Interparental Violence and the Perpetration of Teen Dating Violence. *Child Abuse Negl* 2014; 37: 343–352. 10.1016/j.chiabu.2013.02.001 PMC3670104 23490056

[45] Moylan CA, Herrenkohl TI, Sousa C, et al. The Effects of Child Abuse and Exposure to Domestic Violence on Adolescent Internalizing and Externalizing Behavior Problems. *J Fam Violence* 2011; 25: 53–63. 10.1007/s10896-009-9269-9 PMC2872483 20495613

[46] Abramsky T, Watts CH, Garcia-Moreno C, et al. What factors are associated with recent intimate partner violence? findings from the WHO Multi-country Study on women’s Health and Domestic Violence. BMC Public Health; 11. Epub ahead of print 2011. doi: 10.1186/1471-2458-11-109 21324186 PMC3049145

[47] Kishor S, Johnson K. Profiling domestic violence: A multi-country study. *Stud Fam Plann* 2004; 36: 259–261.

[48] Solanke BL. Does exposure to interparental violence increase women ‘ s risk of intimate partner violence? Evidence from Nigeria demographic and health survey. *BMC Int Health Hum Rights* 2018; 18: 1–13. doi: 10.1186/s12914-018-0143-9 29325549 PMC5765632

[49] Ackerson LK, Kawachi I, Barbeau EM, et al. Effects of individual and proximate educational context on intimate partner violence: A population-based study of women in India. *Am J Public Health* 2008; 98: 507–514. doi: 10.2105/AJPH.2007.113738 18235066 PMC2253590

[50] Yakubovich AR, St H, Murray J, et al. Risk and Protective Factors for Intimate Partner Violence Against Women: Systematic Review and Meta-analyses of Prospective–Longitudinal Studies. *AJPH Res* 2018; 108: 1–11. doi: 10.2105/AJPH.2018.304428 29771615 PMC5993370

[51] Patel R, Gupte SS, Srivastava S, et al. Experience of gender-based violence and its effect on depressive symptoms among Indian adolescent girls: Evidence from UDAYA survey. *PLoS ONE* 2021; 16: 1–18. doi: 10.1371/journal.pone.0248396 33765009 PMC7993765

[52] Carlyle KE, Slater MD, Chakroff JL. Newspaper Coverage of Intimate Partner Violence: Skewing Representations of Risk. *J Commun* 2008; 58: 1–17. doi: 10.1111/j.1460-2466.2007.00379.x 21297889 PMC3032440

[53] Decker MR, Peitzmeier S, Olumide A, et al. Prevalence and health impact of intimate partner violence and non-partner sexual violence among female adolescents aged 15–19 years in vulnerable urban environments: A multi-country study. *J Adolesc Health* 2014; 55: S58–S67. doi: 10.1016/j.jadohealth.2014.08.022 25454004

